# Dialysate copeptin and peritoneal transport in incident peritoneal dialysis patients

**DOI:** 10.1007/s11255-019-02191-5

**Published:** 2019-06-11

**Authors:** Maciej Fijałkowski, Krzysztof Safranow, Bengt Lindholm, Kazimierz Ciechanowski, Anna Maria Muraszko, Barbara Dołęgowska, Katarzyna Dołęgowska, Edyta Golembiewska

**Affiliations:** 1 Department of Nephrology, Transplantology and Internal Medicine, Pomeranian Medical University, Al. Powstancow Wielkopolskich 72, 70-111 Szczecin, Poland; 2 Department of Biochemistry and Medical Chemistry, Pomeranian Medical University, Szczecin, Poland; 3 Division of Renal Medicine and Baxter Novum, Department of Clinical Science, Intervention and Technology, Karolinska Institutet, Campus Flemingsberg, Stockholm, Sweden; 4 Department of Laboratory Diagnostics and Molecular Medicine, Pomeranian Medical University, Szczecin, Poland

**Keywords:** Chronic kidney disease, Copeptin, Peritoneal dialysis, Solute transport

## Abstract

**Purpose:**

Systemic and intraperitoneal inflammation are characteristic features of patients with end-stage renal disease undergoing chronic peritoneal dialysis (PD). Arginine vasopressin (AVP) and its surrogate marker copeptin play important roles in many pathophysiological processes in chronic kidney disease. The aim of this study was to assess if copeptin concentrations in plasma and dialysate were related to peritoneal transport parameters and residual renal function (RRF) in incident PD patients.

**Methods:**

In 37 clinically stable incident PD patients (mean age 50 years, 68% women, 32% diabetes), a 4 h peritoneal equilibration test (PET) was performed 4–6 weeks after the onset of PD. Plasma (at 2 h of PET) and dialysate (at 4 h) concentrations of copeptin, high-sensitivity C-reactive protein and interleukin-6 (IL-6) were determined.

**Results:**

Plasma (80.7 ± 37.3 pg/mL) and dialysate (33.2 ± 18.0 pg/mL) concentrations of copeptin were correlated (*R*s = 0.52, *p* = 0.001). Plasma and dialysate copeptin concentrations were negatively correlated with renal function as assessed by renal Kt/V (*R*s = − 0.38; *p* = 0.021 and *R*s = − 0.33; *p* = 0.047, respectively). At PET, dialysate copeptin negatively correlated with D/P creatinine (*R*s = − 0.35, p = 0.033), and positively with D/D0 glucose (*R*s = 0.33, *p* = 0.045) and ultrafiltration (*R*s = 0.37, *p* = 0.024). Multivariate analysis showed that low dialysate copeptin (*β* = –0.30, *p* = 0.049) and high dialysate IL-6 (*β* = + 0.40, *p* = 0.012) were independent determinants of higher D/P creatinine.

**Conclusions:**

Dialysate copeptin was negatively associated with D/P creatinine in incident PD patients suggesting a potential influence of copeptin or AVP on peritoneal solute transport rate that might involve vasoactive mechanisms.

## Introduction

Chronic kidney disease (CKD) affects millions of people worldwide and end-stage renal disease (ESRD) patients have a high risk of premature cardiovascular death. Apart from traditional risk factors such as high age, smoking, hypertension and/or diabetes, the presence of persistent low-grade systemic inflammation with increased concentration of pro-inflammatory cytokines has been acknowledged as an important underlying mechanism of cardiovascular disease in ESRD [1–4]. The causes of inflammation include comorbidities other than ESRD, decreased renal function with retention of uremic toxins, imbalance between increased production and reduced clearance of pro-inflammatory cytokines, increased oxidative stress, and the dialysis procedure itself.

Peritoneal dialysis (PD), a method of renal replacement therapy in which the patient’s peritoneum is used as a membrane through which water and solutes are exchanged between blood and dialysate, may contribute to intraperitoneal inflammation due to the bioincompatibility of dialysis fluids, episodes of PD-related peritonitis, and catheter infections [5, 6]. Local peritoneal inflammation has been linked to higher peritoneal solute transport rate (PSTR) as assessed by dialysate/plasma (D/P) ratio of creatinine in peritoneal equilibration test (PET) [7]. There is an ongoing search for biomarkers associated with peritoneal transport rate; so far, the dialysate concentration of interleukin-6 (IL-6) has been found to be strongly related to D/P ratio of creatinine [2, 8].

Copeptin is a 39-amino-acid glycosylated peptide of molecular mass approximately 5 kDa, synthesized in the hypothalamus as a C-terminal part of pre-pro-hormone of arginine vasopressin (AVP) [9, 10]. It is difficult to measure the concentration of AVP in the blood due to its instability and short half-life. As copeptin and AVP are released in equimolar amounts and copeptin is stable for more than 24 h after blood withdrawal, copeptin is increasingly used as an easily measured surrogate marker of vasopressin [11]. However, the biologic role of copeptin in circulation is still unknown. High circulating concentrations of copeptin have been linked to a decline in glomerular filtration rate (GFR) and greater risk of new-onset CKD [12]. Moreover, copeptin has been reported to be associated with increased risk of cardiovascular complications such as hypertension, myocardial infarction, and heart failure but also with inflammatory response in sepsis [13, 14]. However, it is not known to what extent copeptin and AVP may influence peritoneal transport.

The aim of this study was to assess if copeptin concentrations in plasma and dialysate are related to peritoneal dialysis transport parameters and residual renal function (RRF) in incident PD patients.

## Methods

### Patients

Thirty-seven incident peritoneal dialysis patients at Peritoneal Dialysis Center, Department of Nephrology, Transplantology and Internal Medicine, Szczecin, Poland, were enrolled in the study 4–6 weeks after the dialysis onset. All patients were started on continuous ambulatory peritoneal dialysis (CAPD) using four 2 L 1.36% glucose-based dialysates with calcium concentration of 1.25 mmol/L (Baxter Healthcare, or Fresenius Medical Care). The patients were clinically stable and did not present signs or symptoms of overt infection or malignancy. None of the patients had peritonitis at the time of the study or in the 4 weeks preceding the study. All patients gave written informed consent. The study was approved by the local Bioethics Committee.

The charts of the patients were reviewed and their age, gender, weight (measured when dialysate was drained out), body mass index (BMI), type of nephropathy, presence of diabetes, residual renal function (RRF), and use of antihypertensive drugs were recorded.

The main characteristics of the study group are presented in Table 1. The causes of ESRD were as follows: diabetes (12), chronic glomerulonephritis (10), hypertension (5), autosomal dominant polycystic kidney disease (3), chronic pyelonephritis (2), and unknown (5). The study group consisted of ‘early start’ patients with relatively high residual renal function and total Kt/V.

**Table 1:** Characteristics of study population

General characteristics	
Age (years)	50.4 ± 13.8
Gender (males/females) (%)	12/25 (32/68)
BMI (kg/m^2^)	24.4 ± 3.7
Diabetes (*n*) (%)	12 (32.4)
Hypertension (*n*) (%)	35 (94.6)
Use of loop diuretics (*n*) (%)	29 (78.4)
Use of ACE inhibitors (*n*) (%)	16 (43.2)
Use of ARB (*n*) (%)	5 (13.5)
Diuresis (mL/day)	1849 ± 936
**Biochemical data**	
Blood hemoglobin (g/L)	114.68 ± 11.45
Serum total protein (g/L)	63.24 ± 6.08
Serum albumin (g/L)	36.11 ± 3.86
Serum creatinine (mg/dL)	4.6 ± 2.04
Serum urea (mg/dL)	90.2 ± 30.7
Serum total calcium (mmol/L)	2.29 ± 0.19
Serum total phosphorus (mmol/L)	1.39 ± 0.33
Serum parathormone (PTH) (pg/mL)	261 ± 197
Plasma hsCRP (μg/mL)	4.1 ± 3.9
Dialysate hsCRP (μg/mL)	0.2 ± 0.1
Plasma IL-6 (pg/mL)	6.6 ± 7.5
Dialysate IL-6 (pg/mL)	30.7 ± 24.6
Plasma copeptin (pg/mL)	80.7 ± 37.3
Dialysate copeptin (pg/mL)	33.2 ± 18.0
**Peritoneal dialysis parameters**	
Daily dialysate (mL)	8354 ± 1606
PET UF (mL)	645 ± 275
Dialysate-to-plasma (D/P) creatinine ratio at 4 h in PET	0.70 ± 0.09
D/D0 glucose ratio in dialysate in PET	0.27 ± 0.09
Peritoneal *Kt*/*V*	1.4 ± 0.4
Renal *Kt*/*V*	1.8 ± 0.9
Total *Kt*/*V*	3.2 ± 1.0

Data are shown as arithmetic mean ± SD or as number (percent)

*BMI* body mass index, *hsCRP* high-sensitivity C-reactive protein, *IL-6* interleukin 6, *PET* peritoneal equilibration test, *D/P* dialysate-to-plasma ratio of creatinine at 4 h, *D/D0* ratio of dialysate glucose at 4 h’ dwell time to dialysis glucose at 0 dwell time, *UF* ultrafiltration, *Kt/V* urea clearance ratio, *K* total dialysate and urinary urea clearance, *t* time, *V* patient’s volume of distribution of urea

### Procedure

A fasting blood sample was collected from each patient. Measurements of plasma urea, creatinine, albumin, high-sensitivity C-reactive protein (hsCRP), and IL-6 were performed using routine laboratory techniques on Architect c8000, Abbott.

Copeptin concentration in EDTA-anticoagulated blood plasma and in dialysate was measured with a commercial enzyme immunoassay (Cloud-Clone Corp., Houston, USA) according to the manufacturer's protocol. The minimum detectable concentration of copeptin is typically < 6.1 pg/mL. The intra-assay coefficient of variation (CV) was < 10% and the inter-assay CV was < 12%.

PET test was performed with 3.86% glucose dialysate as standard. Three dialysate samples were obtained during the test: immediately after completion of the fluid instillation and after 2 and 4 h. Blood samples were obtained 2 h after dialysate instillation. D/P for creatinine and D/D0 for glucose were calculated. Dialysate concentrations of copeptin, high-sensitivity C-reactive protein, and interleukin-6 (IL-6) at 4 h were determined.

24-hour dialysate effluent and urine were also collected for measurement of Kt/V.

### Statistical analysis

Spearman’s rank correlation coefficient (*R*s) was used to measure associations between quantitative variables. Mann–Whitney’s test was used to compare values between groups. General linear model (GLM) was used for multivariate analysis, with prior logarithmic transformation of variables with non-normal distribution. *p* value < 0.05 was considered statistically significant. Statistica 13 software was used for the statistical analysis.

## Results

**Fig. 1:**
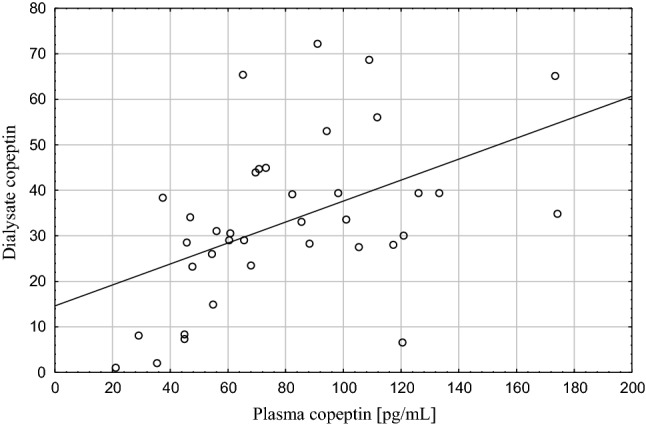
Association of plasma and dialysate copeptin

Mean plasma and dialysate copeptin concentrations are shown in Table 1. There was a strong positive correlation between the plasma and dialysate concentrations of copeptin (*R*s = 0.52, *p* = 0.001), see Fig. 1.

### Relationship between copeptin and clinical features

Plasma copeptin did not differ between men and women (80.44 ± 44.93 vs. 80.76 ± 34.04 pg/mL, respectively) and there was no association with age and diabetes mellitus (*p* > 0.8). In univariate analysis, plasma copeptin concentrations were significantly positively associated with plasma urea (*R*s = 0.38; *p* = 0.019), negatively with renal *Kt*/*V* (*R*s = − 0.38; *p* = 0.02) and total *Kt*/*V* (*R*s = − 0.36; *p* = 0.03), but no association with peritoneal *Kt*/*V* was found (*R*s = 0.01; *p* = 0.96). The association between plasma copeptin and 24-h diuresis was negative, albeit of borderline significance (*R*s = − 0.32, *p* = 0.06). Associations of plasma and dialysate copeptin with selected clinical features and laboratory parameters in incident PD patients are presented in Table 2.

**Table 2:** Associations of plasma and dialysate copeptin with selected clinical features and laboratory parameters in incident PD patients

Correlated parameters	Plasma copeptin	Dialysate copeptin
Age	0.22	0.35*
BMI	0.02	0.01
Serum albumin	0.21	0.23
Serum creatinine	0.21	0.02
Serum urea	0.38*	0.30
Plasma hsCRP	0.08	0.10
Dialysate hsCRP	0.26	− 0.08
Plasma IL-6	0.28	0.20
Dialysate IL-6	− 0.01	− 0.13
Total *Kt*/*V*	− 0.36*	− 0.33*
Renal *Kt*/*V*	− 0.38*	− 0.33*
Peritoneal *Kt*/*V*	0.01	− 0.06
Diuresis	− 0.31	− 0.24

Spearman *R*s values are presented

*BMI* body mass index, *hsCRP* high-sensitivity C-reactive protein, *IL-6* interleukin 6, *Kt/V* urea clearance ratio, *K* total dialysate and urinary urea clearance, *t* time, *V* patient’s volume of distribution of urea

*p < 0.05

### Relationship between copeptin and peritoneal transport parameters

**Fig. 2:**
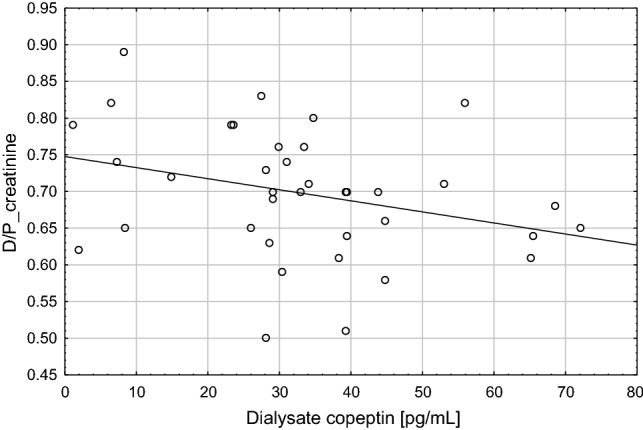
Association of dialysate copeptin and D/P creatinine

**Fig. 3:**
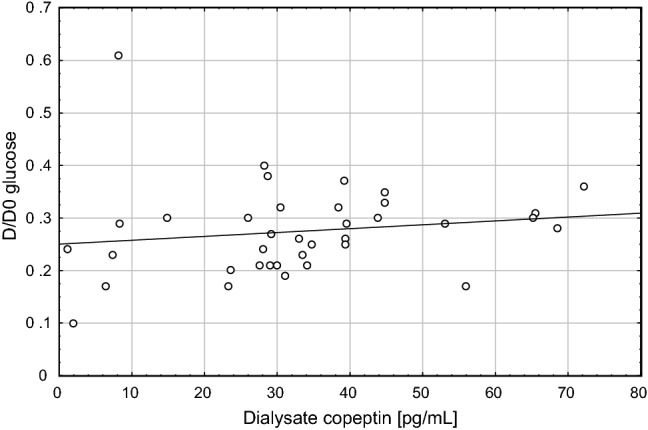
Association of dialysate copeptin and D/D0 glucose

Correlations of peritoneal equilibration test (PET) parameters with clinical and laboratory parameters including plasma and dialysate copeptin are presented in Table 3. There was a negative association (*R*s = − 0.35; *p* < 0.05) between dialysate copeptin and D/P creatinine, as shown in Fig. 2, and positive association (*R*s = 0.33; *p* < 0.05) between dialysate copeptin and D/D0 glucose, as shown in Fig. 3.

**Table 3:** Correlations of peritoneal equilibration test (PET) parameters with clinical and laboratory parameters including plasma and dialysate copeptin

Correlated parameters	PET UF	D/P creatinine	D/D0 glucose
Age	− 0.09	− 0.09	− 0.10
BMI	− 0.02	− 0.17	− 0.04
Hemoglobin	− 0.17	− 0.01	0.13
Serum albumin	0.15	− 0.31	0.29
Serum creatinine	0.21	0.05	0.07
Plasma copeptin	0.09	0.10	− 0.12
Dialysate copeptin	0.37*	− 0.35*	0.33*
Plasma hsCRP	− 0.27	0.01	0.002
Dialysate hsCRP	− 0.37*	0.28	− 0.18
Plasma IL-6	− 0.10	0.13	− 0.18
Dialysate IL-6	− 0.11	0.44**	− 0.58***

Spearman *R*s values are presented

*BMI* body mass index, *hsCRP* high-sensitivity C-reactive protein, *IL-6* interleukin 6, *PET* peritoneal equilibration test, *D/P* dialysate-to-plasma ratio of creatinine at 4 h, *D/D0* ratio of dialysate glucose at 4 h’ dwell time to dialysis glucose at 0 dwell time, *UF* ultrafiltration (ml)

*p < 0.05, **p < 0.01, ***p < 0.001

### Multivariate analysis

Since univariate analysis showed that copeptin and IL-6 dialysate concentrations were most strongly associated with D/P creatinine, multivariate analysis was performed with D/P creatinine as dependent variable and dialysate copeptin and logarithmically transformed dialysate IL-6 concentration as independent variables. GLM showed that low dialysate copeptin (*β* = − 0.30, *p* = 0.049) and high dialysate IL-6 (*β* =+ 0.40, *p* = 0.012) are independent significant determinants of higher D/P creatinine in patients at the onset of PD. When multivariate analysis was performed with dialysate copeptin as dependent variable and age, ultrafiltration in PET, and plasma copeptin as independent variables, GLM showed that higher age (*β* = 0.28, *p* = 0.040), high ultrafiltration in PET (*β* = 0.40, *p* = 0.005), and high plasma copeptin (*β* = 0.41, *p* = 0.004) are independent significant determinants of higher dialysate copeptin.

Multivariate analysis with D/D0 glucose as dependent variable and dialysate copeptin, ultrafiltration in PET, and logarithmically transformed dialysate IL-6 concentration as independent variables showed that only low dialysate IL-6 (*β* = − 0.60, *p* = 0.001) is an independent significant determinant of high D/D0 glucose.

## Discussion

Our study is the first to report that dialysate copeptin, a surrogate marker for AVP, is associated with peritoneal transport assessed by PET in PD patients who were investigated 4–6 weeks following initiation of PD therapy. A main target for active AVP is in the kidneys, where it increases aquaporin-mediated water re-absorption from the filtrate, increases permeability to urea by influencing urea transporters, and increases sodium absorption. So far, there is no known biological role for copeptin, and the role of AVP, if any, in peritoneal transport is also not known.

We report that dialysate copeptin was inversely correlated with D/P creatinine, a marker of the transport properties of the capillary wall reflecting the combined impact of vascular surface area (determined by the number of perfused capillaries) and permeability (determined by the number of inter-endothelial small pores). The inverse correlation between dialysate copeptin and D/P creatinine suggest that AVP/copeptin could act as a vasoconstrictive and permeability active agent, or in other ways could influence the transport properties of the peritoneal vasculature. As plasma copeptin did not correlate with D/P creatinine, this appears to be mainly a local effect.

Whereas, so far, studies on copeptin in PD patients are lacking, our results agree with previous observations demonstrating elevated circulating concentrations of copeptin in patients with CKD. Thus, the mean concentration of plasma copeptin in our study, 80.7 ± 37.3 pg/ml (corresponding to 20.07 ± 9.28 pmol/L), was significantly higher than the mean concentration of 4.2 pmol/L in 359 healthy individuals reported by Morgenthaler et al. [10]. While Bhandari et al. in their study found significantly higher plasma copeptin concentration in healthy volunteer males compared to females (4.3 vs. 3.2 pmol/L) [15], we did not observe a difference between the sexes.

The previous studies show that the higher the copeptin concentration, the higher the risk of decline of renal function and progression of CKD [16, 17]. Thus, Zittema et al. in their study in IgA nephropathy patients found that a higher level of copeptin was associated with the severity and progression of the disease [18]. Similar results were obtained in studies in patients with autosomal dominant polycystic kidney disease (ADPKD) [19, 20].

Plasma copeptin levels are increased also in hemodialyzed patients and in kidney transplant recipients [21, 22]. In hemodialyzed subjects, the concentration of copeptin positively correlates with pre-dialysis body fluid volume [22]. In the study by Artunc et al., plasma copeptin showed a positive correlation with time on dialysis and a negative correlation with residual diuresis [23]. This is in consistence with our study which showed an inverse correlation between plasma copeptin and residual diuresis. Moreover, Ettema et al. showed that in hemodialyzed patients, plasma copeptin levels rise during hemodialysis and that clearance of vasopressin by HD was higher than that of copeptin [24, 25]. On the other hand, Rasche et al. showed that plasma copeptin levels, which were approximately 11-fold higher in hemodialyzed patients compared to normal population, decrease during the hemodialysis (HD) session. The relative reduction of copeptin during HD correlated inversely with the Kt/V ratio, indicating that copeptin might be eliminated by HD [26].

In our study, plasma copeptin correlated with renal Kt/V but not with peritoneal Kt/V, suggesting that kidneys play a major role in the elimination of copeptin. Similar to Rasche et al. [26], we did not find a significant association of copeptin with age or gender.

Our results confirmed the important role of dialysate interleukin-6 concentration, reflecting the intensity of intraperitoneal inflammation, in predicting PSTR, particularly in the early phase of PD treatment [8, 27]. This is in consistence with the results of our study which showed that intraperitoneal IL-6 was associated with PSTR both in univariate and multivariate analyses. It has been found that IL-6 is present in the drained dialysate in higher concentrations that can be explained by diffusion from plasma, implying its local production [28]. In the present study, the concentration of dialysate IL-6 was almost fivefold higher that of plasma IL-6.

In contrast, the higher concentration of plasma as compared to dialysate copeptin and the correlation between dialysate and plasma copeptin (Fig. 1) suggest that dialysate copeptin is mainly stemming from transport of circulating copeptin into the dialysate. Dialysate copeptin showed a negative correlation with PSTR (Fig. 2) and a positive correlation with peritoneal ultrafiltration—which is inversely related to PSTR. We speculate that the mechanisms of this phenomenon might involve increased transport of copeptin from blood to dialysate due to convective transport through the pores of the endothelium with the higher ultrafiltration flow and, in addition, the impact of vasoconstriction, leading on the one hand to a reduction of the number of perfused microcapillaries, but, on the other hand, to increased hydrostatic pressure—a driving force for increased solute transport—in the capillaries of the peritoneal tissue. Although dialysate copeptin was associated with D/D0 glucose in univariate analysis, these different mechanisms, including not only solute transport through small pores, but also convective transport and ultrafiltration might be the reason that dialysate copeptin did not turn out to be the significant determinant of D/D0 glucose in multivariate analysis. Vasopressin contributes to vasoconstriction by different mechanisms: stimulation of the renin–angiotensin–aldosteron system (RAAS), direct effect on smooth muscle cells, or via secretion of endothelin-1 (ET-1) from endothelial cells, which aggravates endothelial dysfunction [13, 29, 30]. Kocyigit et al. in a study in ADPKD patients found that copeptin might be considered as a marker of endothelial dysfunction [31]. The influence of vasopressin administered intravenously on peritoneal membrane was examined in dogs; AVP infusion led to the decrease in dialyzed urea, which was interpreted as a decrease in the total membrane area available for diffusion caused by the decrease in splanchnic blood flow [32]. Vasoactive changes in peritoneal membrane vessels lead to changes in PSTR [33]. The reason for the finding in the present study that dialysate copeptin—and not plasma copeptin—was associated with peritoneal transport parameters is unclear.

The results of the present study should be considered given some important limitations. First, the examined group of patients is relatively small and investigations were carried out during the initial weeks of patients starting PD therapy. Second, given the cross-sectional character of the study, the cause–effect relation cannot be determined. Thirdly, it is not clear if or to what extent copeptin as such may act on the peritoneal vasculature, and AVP was not measured. Further studies are needed to elucidate the role of copeptin in the function of peritoneal membrane in PD patients.

In summary, we report that an increased concentration of dialysate copeptin, a surrogate marker of AVP, associates with two markers of PSTR, i.e., inversely with D/P creatinine and positively with D/D0 glucose, and with increased peritoneal ultrafiltration during PET, while no such associations were observed for plasma copeptin. Whether these findings reflect changes associated with local effects of AVP or if dialysate copeptin per se may influence PSTR is unclear and should be explored in future studies involving larger number of patients.
